# Molecular Pathophysiological Mechanisms in Huntington’s Disease

**DOI:** 10.3390/biomedicines10061432

**Published:** 2022-06-17

**Authors:** Anamaria Jurcau

**Affiliations:** 1 Department of Psychoneurosciences and Rehabilitation, Faculty of Medicine and Pharmacy, University of Oradea, 410073 Oradea, Romania; anamaria.jurcau@gmail.com; 2 Neurology 3 Ward, Clinical Emergency County Hospital Oradea, 410169 Oradea, Romania

**Keywords:** Huntington’s disease, mutant huntingtin, animal models, excitotoxicity, transcriptional dysregulation, proteostasis, mitochondrial dysfunction, oxidative stress, brain-derived neurotrophic factor

## Abstract

Huntington’s disease is an inherited neurodegenerative disease described 150 years ago by George Huntington. The genetic defect was identified in 1993 to be an expanded CAG repeat on exon 1 of the huntingtin gene located on chromosome 4. In the following almost 30 years, a considerable amount of research, using mainly animal models or in vitro experiments, has tried to unravel the complex molecular cascades through which the transcription of the mutant protein leads to neuronal loss, especially in the medium spiny neurons of the striatum, and identified excitotoxicity, transcriptional dysregulation, mitochondrial dysfunction, oxidative stress, impaired proteostasis, altered axonal trafficking and reduced availability of trophic factors to be crucial contributors. This review discusses the pathogenic cascades described in the literature through which mutant huntingtin leads to neuronal demise. However, due to the ubiquitous presence of huntingtin, astrocytes are also dysfunctional, and neuroinflammation may additionally contribute to Huntington’s disease pathology. The quest for therapies to delay the onset and reduce the rate of Huntington’s disease progression is ongoing, but is based on findings from basic research.

## 1. Introduction

Although descriptions of the movement disorder named by Paracelsus (1493–1541) as “chorea” can be found as early as 1374, a comprehensive description of Huntington’s disease was given by George Huntington in an entirely unreferenced paper called *On Chorea*, published in the Medical and Surgical Reporter of Philadelphia on 13 April 1872 in volume 26, no. 15 [1]. The 22-year-old physician very suggestively also described the mode of transmission that will later be known as autosomal dominant with complete penetrance in the following words: “When either or both of the parents have shown manifestations of the disease… one or more of the offspring almost invariably suffer from the disease, if they live to adult age. However, if by any chance, these children go through life without it, the thread is broken and the grandchildren and great-grandchildren of the original shakers may rest assured that they are free from the disease” [2].

The search for the genetic defect began in the 1980s in a small community living around Lake Maracaibo in Venezuela, where Dr. Amerigo Negrette diagnosed many cases of Huntington’s disease (HD) in the 1950s [1]. After identifying through linkage analyses a polymorphic deoxyribonucleic acid (DNA) marker on the fourth chromosome in HD patients [3], the Huntington Disease Collaborative Research Group linked the disease to the IT15 gene (“interesting transcript 15”, subsequently renamed as the huntingtin gene), whose first exon contained a repetitive unstable CAG (cytosine-adenine-guanine) sequence, which was significantly expanded in HD patients [4]. 

## 2. Genetics of Huntington’s Disease

The CAG repeat contained in the first exon of the huntingtin gene varies even in healthy individuals, ranging from 6 to about 25 [1]. Individuals having between 27 and 35 CAG repeats do not show symptoms of HD, but are at risk of transmitting the disease to their offspring due to a phenomenon known as “genetic anticipation” [5]. The risk of expansion of the unstable sequence of CAG repeats during mitosis is higher in spermatogenesis compared to oogenesis [6], which explains why patients with paternal inheriting of the disease have longer CAG repeats and develop symptoms earlier [5].

Individuals having ≥ 40 CAG repeats will develop the disease, and the larger the trinucleotide repeat, the earlier the onset of HD; patients having ≥60 CAG repeats develop juvenile-onset HD [7,8]. However, the CAG repeat number explains only 40–50% of the variance in age of onset and disease severity. A number of other genetic factors contribute to the rest of the variance, such as:

- The CAG repeat size in the normal allele [1,9].
- The presence of the Δ2642 glutamic acid polymorphism (a deletion of three nucleotides in the codon encoding glutamic acid) [10].
- Polymorphisms in the gene encoding for the glutamate receptor GluR6 [11].
- Polymorphisms in the gene encoding the N-methyl-D-aspartate (NMDA) receptor subunit 2B [12].
- Genetic variations of the PPAR-γ (peroxisome proliferator-activated receptor gamma) coactivator 1α (PGC-1α) [13].
- Polymorphisms of the genes encoding for apoptosis signal-regulating kinase 1 (ASK1) and mitogen-activated protein kinase 6 (MAP2K6) [14], or for genes related to mitochondrial activity, such as the CHCHD2 mitochondrial-related neurodegenerative disorder risk gene [15].
- Genes that colocalize with the expanded CAG repeats, and are more likely to be replicated, such as the G-protein-coupled receptor (GPCR) 161 allele [16], which has been shown to be involved in DNA repair processes.

Additionally, the HD-MAPS study identified several other potential loci that may contain genes able to modify the age of onset in HD, located at chromosomes 4p16, 4p16.3, 6q24-26, or 6p21-23, and which deserve further investigation [17]. Genome-wide association studies (GWAS) and transcriptome-wide association studies (TWAS) continue to identify genetic modifiers of the age of onset and course of HD, which could be potential therapeutic targets [18,19,20]. 

## 3. Models of HD

Research on a disease is facilitated by generating cellular and animal models of the disease. 

### 3.1. Chemical Models

Before the identification of the genetic defect, animal models of HD were obtained by intrastriatal injections of kainic acid, or quinolinic acid to rodents [21], suggesting that excitotoxicity may play a role in pathogenesis, or of mitochondrial toxins such as 3-nitropropionic acid [22], supporting the implication of mitochondrial dysfunction in the pathogenesis of the disease. Although these models replicated the regional neuronal loss seen in HD, they did not allow the identification of the pathophysiological mechanisms induced by the mutant protein [1] or the study of the progression of the disease [23].

### 3.2. Genetic Animal Models

After the discovery of the HD gene, several genetic animal models of HD were engineered, starting with inserting fragments containing the human huntingtin promoter and exon 1 of the human huntingtin gene into the animal genome [24,25]. 

#### 3.2.1. Fragment Transgenic Mouse Models

The R6 line of transgenic mice is one of the most widely used models, with the R6/2 line having a 1.9 kb fragment inserted, which contains the human huntingtin promoter and exon 1 of the human huntingtin gene with 144 CAG repeats [1], while the R6/1 transgenic mouse model contains about 110 CAG repeats [24]. Although the second model exhibits a more protracted disease course, both models display a rather aggressive and rapidly progressing phenotype early in life, resembling the juvenile form of human HD [26]. 

Another transgenic mouse line, the N171-83Q line, has 82 CAG repeats under the control of the mouse prion protein promoter, and expresses 171 amino acids of the human huntingtin protein only in neurons [27]. The animals have a later onset of symptoms, allowing the study of presymptomatic therapies [1].

The conditional mouse models of HD, such as the one described by Yamamoto et al., in which the huntingtin exon 1 containing 94 CAG repeats could be silenced by doxycycline and activated by doxycycline removal, allowed for more refined research regarding the events occurring by silencing the mutant gene [28].

#### 3.2.2. Full-Length Transgenic Mouse Models

Full-length huntingtin-expressing transgenic mice show neuronal loss and a gradual development of symptoms more similar to the human disease as compared to the NH_2_-terminal fragment models [1]. A group of researchers at the University of British Columbia created several yeast artificial chromosomes (YAC) expressing normal (YAC18) and mutant human huntingtin (YAC46, YAC72, and YAC128) lines of transgenic mice [29], which exhibited age-dependent striatal and cortical neurodegenerative features, as well as progressive motor and cognitive deficits [30]. 

Another full-length model of HD using a bacterial artificial chromosome (BAC) carrying 97 CAG repeats [31] has given insight into the role of cell–cell interactions in HD pathogenesis [32,33].

#### 3.2.3. Knock-In Mouse Models

These models were produced by introducing CAG repeats (from 48 to 200) into the endogenous mouse HD gene on chromosome 5 [23,34] and have the advantage of expressing the full-length mutant protein in its native genomic context [23]. These animals exhibit subtle behavioral changes at onset followed by motor abnormalities later in life and, thus, more closely mimic the human disease [34].

#### 3.2.4. Other Animal Models of HD

Rat models of HD are mostly used for evaluating therapeutic strategies because of larger sets of available behavioral tests that allow the identification of neurological deficits [1]. Widespread expression of mutant huntingtin has been achieved with the help of a rat HD complementary DNA fragment carrying a 51 CAG expansion under the control of the rat HD promoter [35]. 

Miniature pig, sheep, as well as non-human primate models of HD are also available [1,36,37] and have the advantage of brain size and physiology more closely resembling the human one, thereby being suitable for preclinical experiments and safety studies [1]. 

#### 3.2.5. Non-Mammalian Models of HD

Genetically manipulating animals is costly, time-consuming, and may raise ethical issues. The molecular mechanisms of HD can be studied also in cheaper, faster, and more simple models, such as the *Drosophila melanogaster* model, in which engineered foreign genes can be expressed in tissue-specific and temporal regulated patterns leading to progressive loss of motor function [38]. *Caenorhabditis elegans* and *Danio rerio* (zebrafish) are also suitable for genetically modeling and performing molecular research on HD [39].

#### 3.2.6. Cell Lines for In Vitro Studies

Recently, in vitro experiments came to be performed on several HD patient-derived cell lines, such as fibroblast-derived cell lines or pluripotent stem cell lines, which can be differentiated into neural precursor cells [40,41]. In an attempt to overcome the limitations of two-dimensional culture experiments and to mimic the intercellular signaling and circuitry of the human brain, cerebral organoids (“minibrains”) derived from patient-induced pluripotent stem cells were developed and are useful tools in research [42,43].

## 4. Normal and Mutant Huntingtin

The gene encoding for huntingtin is an evolutionary old gene [44], found in old deuterostomes [45] and across vertebrate species [1]. The gene product, huntingtin protein, is expressed ubiquitously in humans and rodents, the highest levels being found in the central nervous system neurons [46], and appears essential for embryonic development, as embryonic knock-out is lethal [47]. 

### 4.1. Normal Huntingtin Structure

Huntingtin is a 348-kDa protein comprising 3144 amino acids, with a polyglutamine (polyQ) domain at its NH_2_ terminus starting at amino acid position 18 and containing 11–34 glutamine residues [1,2], which forms a polar zipper structure able to bind transcription factors containing polyQ regions [48]. Downstream of the polyQ domain there are several HEAT repeats (**H**untingtin, elongation factor 3 (**E**F3), protein phosphatase 2A (PP2**A**), and the yeast kinase or lipid kinase **T**OR1) organized into 4 clusters [49], which enable the protein to interact with different protein partners [1]. The first 17 NH_2_-terminal amino acids form a membrane-binding domain through which huntingtin (Htt) binds to mitochondria, the endoplasmic reticulum, and the Golgi apparatus [50]. Htt has an α-helical conformation with the HEAT domains connected by smaller bridge domains containing various tandem repeats. It is organized in a complex with Htt-associated protein 40 (HAP40) [51], which has also an α-helical conformation and interacts with Htt through electrostatic forces and hydrophobic bonds, thereby stabilizing the conformation of Htt [52].

### 4.2. Cellular Distribution

Huntingtin is expressed at the highest levels in the neurons of the central nervous system, especially in the pyramidal neurons of cortical layers III and V that project to striatal neurons [53], but also in astrocytes, oligodendrocytes, and microglia [54], as well as in non-neural tissues, such as the muscle fibers [55]. In the cells, Htt and its mRNA associate with the nucleus [56], mitochondria, Golgi apparatus, and endoplasmic reticulum [57,58], and is found in neurites and at synapses interacting with vesicular structures and microtubules [57,59]. 

### 4.3. Post-Translational Modifications

The 17 amino acids of the N-terminal region of Htt are the subject of various post-translational modifications, such as phosphorylation, acetylation, sumoylation, palmitoylation, or other modifications, which influence the aggregation and subcellular localization of the full-length protein and of the N-terminal fragments of Htt [60,61]. Huntingtin can also be ubiquitinated at NH_2_-terminal lysine residues and targeted to proteasomal degradation, thereby maintaining the protein’s homeostasis [62]. The phosphorylation of amino acids, mainly serines, by protein kinase b (Akt), cyclin-dependent kinase 5, or the IκB kinase complex (IKK), is responsible for huntingtin-mediated vesicular transport in neurons, contributes to Htt homeostasis, and appears to be neuroprotective, attenuating aggregate formation [63,64]. Palmitoylation of the protein also contributes to regulating vesicular trafficking [65], while acetylation of lysine residues targets the protein to autophagosomal degradation [66]. Sumoylation modulates the activity, stability, as well as subcellular localization of huntingtin [67]. 

### 4.4. Huntingtin Interacting Proteins

A wide variety of proteins have been shown to interact with Htt, subserving endocytosis, apoptosis, cell signaling, vesicular transport, and regulation of transcription [68,69]. Huntingtin-associated protein 1 (HAP1) interacts with dynactin and, thus, regulates intracellular transport [1,70]. Many of these functions require specific post-translational modifications, which may be impaired in the presence of mutant Htt (mHtt) [71]. Another protein, huntingtin-interacting protein 1 (HIP1) is involved in cytoskeleton assembly and dendritic development [72,73], while binding of Htt to protein kinase C and postsynaptic density 95 (PSD95) regulates synaptic activity [74] and anchoring of postsynaptic glutamate receptors [75]. Through interaction with ADAM10 (A Disintegrin and metalloproteinase domain-containing protein 10), a transmembrane protease, Htt regulates presynaptic neurotransmission, an essential step for the normal functioning of synapses [76,77]. The protein has also an important role in the transcription of many genes. For example, it promotes the expression of brain-derived neurotrophic factor (BDNF) by interacting with the cytoplasmic repressor-element-1 transcription factor/neuron-restrictive silencer factor (REST/NRSF) and preventing the complex from translocating into the nucleus and from activating the silencer element in the promoter of the BDNF gene [78]. The many pathways through which it regulates gene transcription will be detailed in the following section.

### 4.5. Functions of Huntingtin

#### 4.5.1. Huntingtin during Embryonic Development

In embryonic development, huntingtin coordinates cell division, exhibits anti-apoptotic activities in the embryonic ectoderm, and is crucial for the acquisition of the normal neuronal cytoarchitecture [79,80,81]. 

#### 4.5.2. Huntingtin Protects Cells from Apoptosis

Wild-type huntingtin is able to block the activation of caspase-3 and caspase-9 and the formation of the apoptosome, and inhibits the proteolytic activity of caspase-3 by physically interacting with the enzyme [82,83]. In addition, by inhibiting the formation of the HIP1–HIP1-protein interactor complex, it prevents the activation of caspase-8 [84]. 

The protein can activate pro-survival pathways by being a substrate for Akt, and, via the phosphoinositide 3-kinase (PI3K)/Akt pathway stimulates the expression of pro-survival genes and represses the expression of death genes such as Bax and Bcl-2 [85].

#### 4.5.3. Huntingtin and Transcriptional Regulation

The transcription of DNA into messenger RNA (mRNA) is a tightly regulated process involving several transcriptional factors that interact with each other and with regulatory DNA elements of the gene [86].

One transcriptional pathway in which Htt is involved is the cAMP-responsive element (CRE) pathway. The transcription factor CREB (CRE-binding protein) binds to DNA elements that contain CRE in their promoters. The phosphorylation of CREB by protein kinase A allows for the subsequent recruitment of CREB-binding protein (CBP), which remodels chromatin into an open architecture due to its intrinsic histone deacetylase activity. Subsequently, other transcription factors are recruited, leading to the phosphorylation of the carboxy-terminal domain of RNA polymerase II and initiation of transcription [86]. Mutant Htt sequesters CBP in nuclear aggregates preventing it from binding to CRE regions in promoters, thereby impairing transcription [87].

Another transcriptional pathway disrupted in HD is the specificity protein 1 (SP1) pathway. The C-terminal of SP1 contains a DNA binding domain with three zinc finger motifs, while in the N-terminal region there are several glutamine-rich domains that regulate transcriptional activity [88]. The binding of mHtt to the glutamine-rich regions or to the C-terminal domain interferes with the activity of SP1 and may sequester it into nuclear aggregates [89]. 

Wild-type Htt also regulates the activity of genes that contain neuron-restrictive silencer elements (NRSEs) by modulating the nuclear recruitment of NRSE-binding transcription factors [90]. Htt interacts with the REST–NRSF (repressor-element-1 transcription factor–neuron-restrictive silencer factor) in the cytoplasm, reducing its availability for nuclear NRSE-binding sites, thereby promoting the transcription of neuronal genes containing NRSEs, such as the *BDNF* gene. 

#### 4.5.4. The Role of Huntingtin in Axonal and Vesicle Transport

Axonal transport requires intact microtubules, normally functioning motor proteins, correct attachment of cargoes to these proteins, and ATP stores supplied by mitochondria [91]. Htt has an important role in regulating the trafficking of both organelles and vesicles along the axons. Two main motor proteins regulate axonal transport: kinesin binds cargoes mainly for anterograde transport, while dynein is involved mainly in retrograde transport [92,93]. HAP1 interacts with both Htt and the p150-glued subunit of dynactin, a 23-subunit protein complex acting as a co-factor for the microtubule motor cytoplasmic dynein-1 [94]. For mitochondrial trafficking, Miro, a mitochondrial outer membrane protein (a Rho GTPase), plays a pivotal role by being able to form protein complexes with Milton (an adaptor protein), kinesin, and dynein, and thereby to facilitate antero- and retrograde mitochondrial transport [95].

The mechanism through which Htt interferes with the vesicular transport along the axons was studied for BDNF by Saudou and his team [96,97], who showed that Akt-mediated phosphorylation of Htt at serine 421 favors anterograde transport, while dephosphorylation of this amino acid potentiates retrograde transport of BDNF vesicles [98,99]. In addition, recent research has shown that Htt co-localizes with a class of Rab-GTPases-containing vesicles within axons in vivo and that the moving Htt-Rab-containing vesicle uses kinesin-1, possibly kinesin-3, and dynein motors, as well as HIP1 as accessory protein, for its bi-directional movement within axons [100,101]. The axonal transport of synaptic vesicle precursors (SVPs), which contain proteins that are required to fill, dock, and release synaptic vesicles at terminal buttons, regulates, together with endocytosis, the number of synaptic vesicles at the synapses [102]. Htt phosphorylation increases the recruitment of the molecular motor KIF1A on SVPs, promoting anterograde transport and release of the vesicular content at the synapse [70].

#### 4.5.5. Huntingtin and Synaptic Activity

A normal presynaptic function includes the shipping of synaptic vesicles (SVs) from the cell body to the nerve ending [103], the ability to refill SVs with neurotransmitters [104], and the removal of damaged proteins from the presynaptic membrane [105]. ADAM10 is a transmembrane α-secretase that binds together with Htt to piccolo, a cellular matrix protein essential for the maintenance and recycling of SVs [106]. Both Htt and ADAM10 interact with clathrin adaptor protein 2 (AP-2), with Htt serving as a docking protein that helps recruit AP-2 to the presynaptic membrane [107]. As such, loss of function of wild-type Htt due to the polyQ expansion leads to impaired clathrin-mediated SV recycling [108]. Exocytosis of the neuromediator is facilitated through the interaction of Htt with various binding partners, such as HAP1 and HIP14 [109]

The bidirectional transport between the cell soma and postsynaptic membrane of various cargoes is also essential for proper synaptic activity. The Htt/HAP1 complex interacts with kinesin family motor protein 5 (KIF5) and ensures the delivery of GABA_A_ receptors to postsynaptic sites, but the same complex transports α-amino-3-hydroxy-5-methyl-4-isoxazolepropionic acid (AMPA) receptors to postsynaptic sites in excitatory synapses [107]. Htt binds directly to the SH3 domain of PSD95 [110], a component of the membrane-associated guanylate kinase protein family, which anchors the glutamatergic N-methyl-D-aspartate (NMDA), AMPA, and kainate receptors to the postsynaptic density [1,111]. The palmitoylation of PSD95, together with the palmitoylation of AMPA and NMDA receptor subunits, regulates the trafficking and localization of these receptors to the postsynaptic membrane [112]. Impairment of this function of Htt may lead to extrasynaptic NMDAR clustering [113]. Htt also colocalizes with the BDNF receptor TrkB (tyrosine kinase B) at postsynaptic sites. Following activation by BDNF, TrkB is internalized and transported to the cell body, where it exerts pro-survival effects [114]. 

### 4.6. Mutant Huntingtin

The expanded CAG repeats on chromosome 4 result in the formation of a mutant huntingtin protein (mHtt), which, beginning at amino acid 18, contains an expanded polyglutamine (polyQ) stretch with more than 40 glutamine residues [115]. Following proteolysis, the polyQ stretches are released and appear either as soluble cytosolic fragments or form oligomers, fibrillary structures, or aggregates [115]. The altered structure of mHtt modifies the ability to interact with other proteins, leading to a toxic gain of function, or impairs vesicular trafficking and mitochondrial dynamics (loss of function) and ignites a series of pathogenic cascades, which will lead to degeneration of neuronal populations, as detailed below.

## 5. Mechanisms of Neurodegeneration in Huntington’s Disease

The pathology of HD is brain-specific and is characterized by prominent cell loss in the caudate and putamen [116], as well as in other structures of the CNS. The most common grading system of HD pathology was developed by Vonsattel and comprises 5 severity grades of cellular degeneration [117]:

- Grade 0—the brain appears normal on gross examination, but histologically 30–40% of neurons are lost in the head of the caudate nucleus.
- Grade 1—a 50% neuronal loss in the head of the caudate nucleus, with neuronal loss and astrogliosis evident in the tail +/− body of the caudate nucleus.
- Grade 2—striatal atrophy, with ventricular profile of the caudate nucleus less convex than normal.
- Grade 3—severe striatal atrophy with flat ventricular profile of the caudate nucleus.
- Grade 4—atrophy of the striatum and up to 95% neuronal loss, with concave ventricular profile of the caudate nucleus.

The first neurons to degenerate are the striatal medium-sized spiny GABAergic neurons [118]. Other affected neuronal populations are the enkephalin-containing neurons that project to the globus pallidus pars externa, and substance P-containing neurons projecting to the pars reticulata of the substantia nigra. In later stages of the disease, neurons in layers III, IV, and VI of the cortex [116], as well as somatostatin-positive neurons and orexinergic neurons in the hypothalamus degenerate [119,120]. All these neuronal losses are caused by the loss of function and toxic gain of function of the mutant huntingtin protein via diverse pathologic cascades, which will be discussed below. 

### 5.1. Excitotoxicity

Excitotoxicity was the first discussed pathogenic mechanism, stipulating that excessive activation of glutamate receptors by cortical afferents and hypersensitivity of striatal postsynaptic glutamate receptors would contribute to HD pathogenesis via pathological downstream signaling [1]. 

The main source of glutamatergic input to the striatum is the cortex and thalamus [121] Glutamate acts on the postsynaptic membrane via ligand-gated cationic channels (N-methyl-D-aspartate—NMDA, α-amino-3-hydroxy-5-methyl-4-isoxazolepropionate—AMPA, and kainate—KA) as well as via metabotropic receptors coupled to G protein and second messenger systems [122]. The finding that NMDA receptor (NMDAR) agonists better replicated the neuropathological and behavioral manifestations of HD in rodents and primates [23], as well as the fact that NMDAR antagonists could rescue striatal neurons from damage caused by systemic exposure to 3-nitropropionic acid [123], pointed toward a crucial role for NMDARs. Indeed, striatal NMDARs decrease early in human patient HD brains, suggesting that NMDAR-expressing neurons are most vulnerable, but other cerebral regions that contain even higher levels of glutamate receptors, such as the hippocampus, brainstem, or cerebellum, are mostly spared [122]. The selective neuronal vulnerability may be explained by the subunit composition of the NMDARs.

NMDARs are tetrameric complexes formed by two GluN1 (formerly known as NR1) and two GluN2 (or NR2) and/or GluN3 (or NR3) subunits [124]. Four genes encode the GluN2 subunits, resulting in GluN2A-D subunits [23]. The binding of glutamate and allosteric modulator glycine to NMDARs opens a ligand-gated calcium channel, facilitated by the partial depolarization and removal of the magnesium ion blocking the channel pore by glutamate binding to AMPA receptors [125]. The GluN2 subunit, which binds glutamate, is able to change the ion channel permeability and sensitivity to Mg^2+^ and glycine, which binds to the GluN1 and GluN3 subunit [23]. Striatal projection neurons express predominantly GluN2B subunits with small amounts of GluN2A subunits, whereas interneurons predominantly express GluN2D subunits, with low levels of GluN2A and GluN2C, a particularity that might explain the differences in neuronal vulnerability to glutamate toxicity [126]. In addition, reduced transcription of the GluN2B gene and proteolysis of the subunit by calpains in HD alter the functional characteristics of the striatal NMDARs [127], while altered interaction of mHtt with the scaffolding protein PSD95 and tyrosine phosphorylation of NMDARs further sensitizes the receptors to glutamate [110,128]. 

The subcellular localization of NMDARs plays also a critical role in the downstream signaling cascades. The activation of synaptic NMDARs triggers the expression of a variety of anti-apoptotic and pro-survival proteins via phosphorylation of cyclic-AMP responsive element-binding protein (CREB), while the activation of extrasynaptic NMDARs is followed by cellular and mitochondrial calcium overload, increased oxidative stress, dephosphorylation and inactivation of CREB, and promotion of pro-death gene expression [129,130]. In HD, increased calpain activity leads to activation of calcineurin, which dephosphorylates the striatal enriched tyrosine phosphatase (STEP), an enzyme able to dephosphorylate the tyrosine 1472 residue of the GluN2B subunit, leading to reduced synaptic NMDAR expression and lateral diffusion of the GluN2B-containing receptors to extrasynaptic sites [23,127,131,132,133]. 

Additionally contributing to increased stimulation of NMDARs is the deficient activity of the Na^+^-dependent glial transporter of glutamate GLT1, or the equivalent enzyme in mammals and humans, excitatory amino acid transporter 2 (EAAT2), responsible for the removal of extracellular glutamate. Brain samples of HD patients exhibited decreased glutamate transporter mRNA [134] and knockout of GLT1 replicates HD transcriptional dysregulation [135]. 

Moreover, the trafficking of NMDARs is impaired in HD through destabilization of the clathrin-mediated endocytotic complex that involves the NMDA receptors, huntingtin, and HIP1 [113], as well as diverse interactions of post-translationally modified mHtt with PSD95, leading to increased stability of NMDARs at extrasynaptic sites [136]. 

An interesting finding is the fact that glutamate uptake promotes the release of ascorbate, an antioxidant vitamin, in the striatum [137]. Additionally, the huntingtin gene interferes with the translocation of the ascorbate transporter to the plasmalemma, opening the possibility of impaired cellular storage of the antioxidant vitamin in HD [138]. Unfortunately, high dose ascorbate treatment in mouse models only transiently restored the striatal ascorbate levels, but the possibility of glutamate transporter up-regulation with adenoviral vector-mediated or non-viral delivery of the GLT1 or EAAT2 gene is actively pursued [139].

Downstream effects of NMDAR overactivation involve altered intracellular calcium signaling, impaired mitochondrial activity, and triggering of apoptotic pathways, a process exacerbated by the action of mHtt on the mitochondrial transition pore, as will be discussed in Section 5.3. 

Aside from the impaired glutamate transmission in the striate, early alterations in the inhibitory GABAergic transmission that extensively affect the brain in mouse models as well as human HD patients have also been demonstrated recently. GABA is the main inhibitory neuromediator in the brain, which, upon acting on a diversity of receptors, leads to the opening of anion-selective intrinsic channels allowing for chloride anion flow and leading to neuronal hyperpolarization [140]. The GABA receptors are heteropentameric structures composed of a combination of 5 out of 19 subunits (α1-6, β1-3, γ1-3, δ, ε, θ, π, and ρ1-3 subunits) [141]. Synaptic GABA_A_ receptors mediate phasic inhibition and are composed of α1-3 subunits combined with one β and 2 γ subunits, while extrasynaptic GABA_A_ receptors have an α5 and/or a δ subunit substituting a γ subunit and mediate tonic inhibition [142]. Research has shown a decreased binding of benzodiazepines in the caudate nucleus early in the disease progression [140], a finding consistent with the demonstration of a reduced expression of the α2 subunit, the major component of GABA_A_ receptors in the MSNs, and increased expression of the α1 subunit of the receptors demonstrated in mouse HD models [143]. In addition, the expression of α1 and α2 subunits is regulated by GABA_A_ receptor activity and dopamine [144]. Thus, GABAergic receptors undergo complex changes in the striatum, leading to impairments of both synaptic and extrasynaptic GABAergic neurotransmission.

### 5.2. Impaired Proteostasis

Within cells, proteins are continuously synthesized as linear chains of amino acids, which must be folded into the correct three-dimensional structure in order to fulfill their function [145]. The precise balance between protein expression, their correct localization, conformation, and the maintaining of normal concentrations is termed proteostasis [146]. The folding of proteins into their correct conformation is mediated by a series of molecular chaperones (about 90 chaperones described) and about 250 co-chaperones [86,146], which assist in the refolding of proteins when their structure is modified by stress conditions or bind to exposed hydrophobic regions of misfolded polypeptides, thus preventing these residues from aberrantly interacting with other proteins [145]. If protein folding is unsuccessful, the protein must be refolded into its correct structure, or it will be ubiquitylated and targeted for degradation via the ubiquitin–proteasome system (UPS), which acts in both the cytoplasm and nucleus or the lysosome/autophagy pathway, which functions mainly in the cytoplasm [40,93,147]. 

Chaperones, which assist protein folding and disaggregation [148] can be classified according to their molecular weight into: 40 kilodalton heat shock proteins (HSP40s), HSP60s (chaperonins), HSP70s, HSP90s, HSP100, and the small HSPs [149,150]. Many co-chaperones assist chaperones in their tasks, such as the tetratricopeptide repeat (TPR)-domain-containing family (e.g., CHIP, HOP), the BAG (Bcl-2-associated athanogene)-domain-containing family (BAG1–BAG6) and DNAJ-domain-containing HSP40s [151]. 

For UPS-mediated degradation, protein substrates are tagged through polyubiquitination, a process catalyzed by a three-enzyme complex: a ubiquitin-activating enzyme (E1), a conjugating enzyme (E2), and E3 (a ligase). Htt is ubiquitylated by several E3 ligases, such as CHIP (carboxyl terminus of Hsc70-interacting protein), Parkin, WWP1 (WW domain-containing E3 ubiquitin protein ligase 1), or HACE1 (HECT domain and ankyrin repeat-containing E3 ubiquitin-protein ligase 1) [40]. Polyubiquitinated proteins are recognized by the 26S proteasome, a complex formed by one or two 19S regulatory particles and a 20S core particle. The regulatory particle deubiquitinates and unfolds the protein, which is translocated to the catalytic core [152,153,154]. Removal of ubiquitin may also be performed by deubiquitylases (DUBs) but only ATXN3 and USP19 have been shown to deubiquitylate Htt [155,156]. Recent findings suggest that heterotopic polyubiquitin chains may be formed in HD, which can complicate the UPS-mediated degradation of mHtt [157]. 

Because the eukaryotic proteasome cannot digest peptides containing stretches of 9–29 polyglutamine residues [158], misfolded proteins are prone to aggregation and may overwhelm the UPS system [159]. Only puromycin-sensitive aminopeptidase (PSA) is able to digest polyQ fragments, an enzyme abundant in the brain and upregulated in diseases related to polyglutamine expands [160,161]. However, research has shown contradictory results regarding the activity of the UPS system. An interesting finding is that of the more than 300 E3 ligases, the unique α-amine E2 enzyme UBE2W acts on the N-terminus of Htt and that UBE3A specifically targets Htt fragments for ubiquitylation and degradation [40]. The age dependency of UBE3A clearance of Htt in HD knock-in mouse models may explain the late age of onset of symptoms [162]. Compared to normal Htt, mHtt is more susceptible to proteolysis, being cleaved by caspases (caspase-3, -6, -2, and -7), calpains, and aspartyl proteases and generating fragments containing the expanded polyQ stretch that prove toxic and additionally activate the cleaving enzymes in a vicious cycle [1,163]. 

Another essential step in promoting cellular health and maintaining a proper balance between protein synthesis and clearance is autophagy [164], during which lysosomes degrade a portion of the cytosol containing damaged proteins or organelles. Autophagy can be classified into [164,165,166]:

- Macroautophagy, in which a double-membraned vesicle (the autophagosome) forms and fuses with lysosomes, after which their content is degraded by the lysosomal enzymes.
- Microautophagy, a process during which lysosomes wrap around various cytosolic compounds, followed by involution of the membrane and degradation of the vesicle content [167].
- Chaperone-mediated autophagy, a process during which chaperones bind to damaged proteins and to receptors on the lysosomal membrane, leading to translocation of the protein into the lysosome and degradation [168].

Recognition of cargo by autophagosomes, axonal transport, as well as substrate degradation, are all diminished in HD. Rhes, a small GTPase, interacts with Beclin-1 and reduces its inhibitory binding to Bcl-2, but is inactivated by binding to mHtt and sequestered in mHtt aggregates [40]. Being highly expressed in the striatum, Rhes inactivation by mHtt and the subsequent impairment in autophagy may contribute to the selective neuronal damage in the striatum. However, mutant Htt aggregates may also stimulate autophagy by sequestering mTOR (mammalian target of rapamycin), a negative regulator of the autophagic pathway, into the polyQ aggregates [169]. Indeed, increased numbers of autophagosome-like structures have been described in the brains of HD patients by several researchers [170], but many cytosolic autophagosomes are “empty”, carrying no substrate, a situation possibly related to the mHtt-related impairment of cargo recognition by the selective autophagy receptor SQSTM1/p62 [171]. mTOR phosphorylates at Ser757 the autophagy-initiating kinase ULK1 and prevents its phosphorylation by AMPK at Ser317 and Ser777, phosphorylations that activate ULK1. This coordinated regulation of autophagy by phosphorylation depends on the nutrient status of the cell. mTOR signaling prevails in conditions of nutrient sufficiency and AMPK signaling in conditions of glucose starvation. Autophagy induction may compensate for the impaired UPS activity by degrading misfolded mHtt, and increasing autophagy through the overexpression of HDAC6 promotes clearance of mHtt aggregates; although, certain particular conformations may be resistant to autophagy and exert cytotoxic effects [172,173]. In a research setting, overexpression of genes implicated in autophagy or exogenous administration of autophagy activators enhances the clearance of mHtt and improves behavioral abnormalities in mice, while inhibition of autophagy leads to an increased number of aggregates [174]. Increased acetylation of mHtt at lysine residue 444 promotes its trafficking into autophagosomes and improves its clearance [175]. Another protein, LAMP-2A (lysosomal associated membrane protein 2A) is necessary for chaperone-mediated autophagy and the import of mHtt across the lysosomal membrane [64]. The natural decline of LAMP-2A levels with age may also contribute to the onset of HD in older age by enhancing mHtt accumulation [64].

The sequestration of chaperones into mHtt aggregates decreases the amount of available soluble chaperones, enhancing abnormal protein folding [176]. Indeed, the dysfunction of chaperones has been described in HD both in vitro and in animal models. Two aggregation pathways have been described for mHtt. The first pathway involves a nucleated polymerization of polyQ stretches, forming a nucleus, with additional polyglutamine monomers joining the growing aggregate and forming a ribbon-like structure [177]. In the second pathway, oligomers arranged as protofibrils form, comprising the first 17 NH2-terminal amino acids in their core and with the polyQ sequences exposed on their surface [178]. The formation of these inclusions indicates an imbalance between the production and clearance of aggregation-prone proteins, and the number of these inclusions correlates with the length of the polyQ repeats [179].

**Figure 1:**
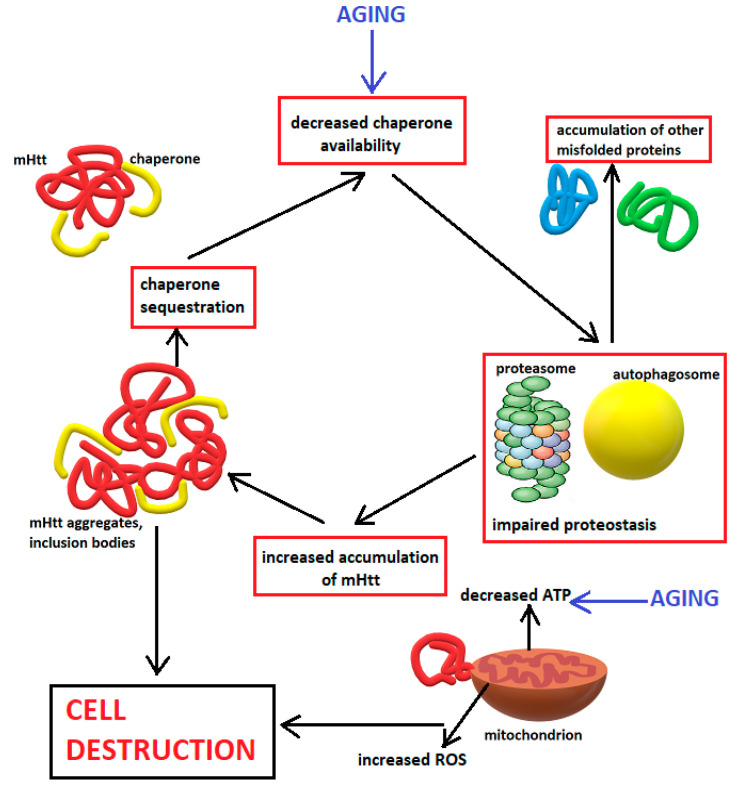
Impaired proteostasis in Huntington’s disease. The chronic production of misfolded huntingtin protein acts synergistically with aging to decrease available chaperones. This leads to accumulation of other misfolded proteins. The mHtt eventually overwhelms the proteasome and impairs the misfolded protein clearance system, an impairment further augmented by the limited availability of ATP due to aging and mHtt-related mitochondrial dysfunction leading to increases in the production of reactive oxygen species (ROS), which, in turn, potentiate mitochondrial dysfunction in a vicious cascade. The accumulation of mHtt promotes its aggregation, further sequestering chaperones in a vicious cycle that disrupts cellular homeostasis and culminates in cell destruction.

Chaperones and co-chaperones, such as CHIP and BAG1, can influence mHtt aggregation form via their interaction with HSP70s [180]. Members of the HSP70 family and their DNAJ-domain-containing HSP40 co-chaperones can inhibit the formation of spherical inclusions, promoting the accumulation of fibrillar, less toxic, aggregates [150]. Another regulator of mHtt aggregation is the TRIC/CCT complex (T-complex protein-1 ring complex, also called CCT for chaperonin containing TCP1), a multi-subunit complex of which the loss of a single subunit can impair the function of the whole complex and accelerate mHtt aggregation [181]. The differential ability of various neuronal types to regulate the ubiquitin–proteasome system (UPS) may contribute to the selective vulnerability of certain neuronal subtypes in HD [182]. In addition, the more pronounced impairment of the UPS in neurons as compared to glia may explain the preferential accumulation of aggregates in neurons and their increased vulnerability [183,184]. Figure 1 provides a schematic representation of the UPS and autophagosome pathways in HD.

However, whether these aggregates are toxic or neuroprotective is still a matter of debate. Favoring the toxic role is the finding that mHtt inclusions correlate with disease progression, as well as the experimental work of Yamamoto and coworkers, who showed in an inducible HD mouse model that turning off mHtt production caused the inclusions to disappear and reversed the behavioral deficits [28]. Moreover, administration of proteasome inhibitors in cellular and animal models of HD increases the number of mHtt aggregates, while overexpression of HSPs reduces mHtt aggregation and increases the lifespan of HD mice [1]. Other experiments suggest rather that mHtt inclusions are a modality through which cells sequester toxic soluble fragments [185], similar to tau pathology, in which soluble oligomers are more toxic than fibrillary tangles [93,186]. It appears that the N17 domain and the polyQ chains promote aggregation [187], while the proline-rich domain inhibits aggregation [188], In addition, huntingtin fibrils with different structures and toxicity can be interconverted [189] and are able to seed neighboring neurons in a prion-like manner [190].

### 5.3. Mitochondrial Dysfunction and Oxidative Stress

Neurons are cells with particularly high energy demands, and rely mainly on mitochondrial oxidative phosphorylation (OXPHOS) to meet these demands. A series of protein complexes of the electron transport chain (ETC) is located on the inner mitochondrial membrane (IMM) and use the electrons removed by reduced nicotinamide adenine dinucleotide (NADH) and flavin adenine dinucleotide (FADH_2_) from the Krebs cycle to pump protons from the matrix into the intermembrane space. The resulting potential gradient across the IMM will be used to synthesize ATP in the final step of OXPHOS [165]. 

In order to supply the necessary energy demands, the number, shape, size, and position in the cytoplasm of mitochondria must be tightly regulated through two opposite processes: mitochondrial fission and fusion [165]. Fission is regulated by dynamin-related/-like protein 1 (Drp1) and dynamin2 (Dnm2) [191]. Cytosolic Drp1 is recruited to the outer mitochondrial membrane (OMM) and bound by adaptor proteins such as mitochondrial fission factor (MFF) and mitochondrial dynamics proteins 49 and 51 (MiD49 and MiD51) [192], followed by recruitment of Dnm2, a GTPase that completes the fission process [193]. Fusion is the opposite process, by which mitochondria fuse allowing for sharing of essential components. The fusion of the OMM is regulated by mitofusins 1 and 2 (Mfn1 and Mfn2) and two GTPases, which hydrolyze the GTP through which the OMM of two adjacent mitochondria are tethered [194], followed by the fusion of the IMM mediated by optic atrophy 1 (OPA1), inserted into the IMM, and which interacts with cardiolipin [195]. A series of post-translational modifications of these proteins can regulate mitochondrial dynamics. MFF phosphorylation enhances Drp1 recruitment and potentiates mitochondrial fission, while phosphorylation of Mfn1 by ERK inhibits mitochondrial fusion and promotes apoptosis [196].

However, given the particular morphology of neuronal cells, mitochondria must also be trafficked along neuronal outgrowths to supply the necessary energy even at distant sites from the cell soma [197]. The core of the complex mediating the trafficking of mitochondria consists of three proteins: the heavy chain of the conventional kinesin-1, Miro, a protein anchored to the outer surface of the mitochondrion (also known as RhoT1 and RhoT2), and milton (also known as TRAK1 and TRAK2), a protein that links kinesin and Miro [198,199]. A key regulator of mitochondrial trafficking is the level of cytosolic calcium concentration. Elevation of cytosolic Ca^2+^ slows down or even stops mitochondrial movement [165]. Another mechanism involves parkin and PINK1 (PTEN-induced kinase 1), which phosphorylate Miro and target it to proteasomal degradation, thereby detaching kinesin from mitochondria [200].

Damaged mitochondria are removed by mitophagy, a specific form of autophagy. After formation of an isolation membrane, the autophagosome, the pre-initiation complex, containing Unc-51-like kinase 1 (ULK1), autophagy-related proteins (Atg) 13 and 101, and focal adhesion kinase family interacting partner 200 (FIP200) is activated [201], followed by recruitment of class III phosphatidyl inositide 3-kinase (PI3K), beclin1, Atg 14, autophagy and beclin 1 regulator (AMBRA1), and vascular protein sorting 34 and 15 (Vps 34 and 15) to produce phosphatidylinositol 3-phosphate (PI3P), also called the initiation complex [202]. Cleavage of pro-LC3 (light chain 3) by Atg4 to LC3-I, further transformed by phosphatidylethanolamine to LC3-II, leads to elongation and closure of the isolation membrane. Υ-aminobutiric acid type A-receptor-associated protein (GABARAP) and GABARAP-like 1 protein (GABARAPL1) presumably play similar roles with LC3 in autophagosome expansion [203]. By phosphorylating Atg13, mTOR prevents its binding to ULK1 and recruitment of FIP200, inhibiting the mitophagy process when growth factors and cellular nutrients are abundant, while starvation inhibits mTOR and ignites mitophagy [204]. The fusion of autophagosomes to lysosomes is mediated by Rab7 and LAMP-2 [205]. Mitochondria can also be extruded from cells and taken up by endocytosis or phagocytosis by neighboring cells and undergo mitophagy, a phenomenon termed transcellular mitophagy [201,206].

Aside from energy production, mitochondria are also essential in cellular calcium homeostasis [207]. Transient fluctuations in the cytosolic Ca^2+^ act as second messengers. Calcium influx occurs through ligand-operated or voltage-gated calcium channels (VGCCs), as well as through release from intracellular Ca^2+^ stores, mainly the endoplasmic reticulum (ER). However, in order for the increases in cytosolic Ca^2+^ to be brief and act as a signal, calcium must be rapidly cleared through calcium efflux, binding to Ca^2+^-buffering proteins, or uptake into the ER or mitochondria [208]. Mitochondrial Ca^2+^ uptake is mediated by voltage-dependent anion-selective channel proteins (VDCAs), which mediate Ca^2+^ transfer into the intermembrane space, and by the mitochondrial Ca^2+^ uniporter (MCU) located on the IMM, which further moves Ca^2+^ into the mitochondrial matrix. Increases in mitochondrial calcium can increase ATP production by activating the electron transport chain (ETC) dehydrogenases [209], but may also lead to the opening of the mitochondrial permeability transition pore (MPTP) and trigger apoptosis through the release of cytochrome c [210].

Finally, mitochondria are important sources of reactive oxygen species (ROS). Even under normal conditions, about 2% of electrons “leak” from the ETC serving to produce ROS. Several mitochondrial proteins and complexes, such as glycerol-3-phosphate dehydrogenase or various cytochrome P450 enzymes in the IMM, monoamine oxidase, located on the OMM, pyruvate dehydrogenase, α-ketoglutarate, and aconitase (enzymes of the tricarboxylic acid cycle) or complexes of the ETC have been shown to contribute to ROS production depending on the mitochondrial membrane potential and activity of the ETC complexes [211]. In turn, mitochondria are a prominent target for ROS, leading to mitochondrial dysfunction and increasing oxidative stress.

A large body of evidence implicates mitochondrial dysfunction and oxidative stress in the pathogenesis of HD. First, F-18 fluorodeoxyglucose PET showed impaired energy metabolism in the caudate, putamen, and cortex even in presymptomatic HD patients [212], while direct measurement of the cellular bioenergetics showed an impaired glycolytic capacity of HD-derived neurons [213]. Patients with HD exhibit a diminished expression of GLUT3 especially in the striatum and cortex [214]. Further, the striatum of HD patients was found depleted of N-acetyl aspartate, with increased production of lactate, suggestive of elevated glycolytic rates [1]. All these findings point toward an important role of mitochondrial dysfunction in HD pathogenesis [215]. 

The cytoplasmic mHtt aggregates can directly interact with mitochondrial proteins and lead to a reduction in the mitochondrial membrane potential [216], as well as a decrease in the activity of complexes I, III, IV, and of the tricarboxylic acid cycle [217]. In addition, postmortem studies of the striatum of HD patients, as well as studies on cultured striatal neurons transfected with mHtt, showed a marked reduction in complex II activity and a depletion of succinate dehydrogenase [215]. These deficiencies, together with the age-dependent decrease in complex I and IV activity [218], increased ROS production and impaired calcium handling [219] and accelerated neuronal degeneration.

Mutant Htt associates with mitochondria-related proteins and directly modulates the permeability of the permeability transition pore, causing cytochrome c release at lower calcium concentrations [220]. Much of the mitochondrial dysfunction appears to be caused by altered nuclear gene transcription mediated by mHtt, in particular of the transcription of the peroxisome proliferator-activated receptor-γ coactivator 1α (PGC1α), a key regulator of energy metabolism involved in glucose metabolism, β-oxidation of fatty acids, and the induction of mitochondrial biogenesis [221]. Mutant Htt prevents the CREB/TAF4 (cAMP responsive element-binding/TATA-binding protein-associated factor 4) complex from binding to the PGC1α promoter [222], thereby inhibiting its expression, and inhibiting the deacetylase activity of Sirt1, which controls CREB/TAF4-mediated transcription through regulation of the acetylation status of TORC1 (transducer of regulated CREB activity 1) [223].

**Figure 2:**
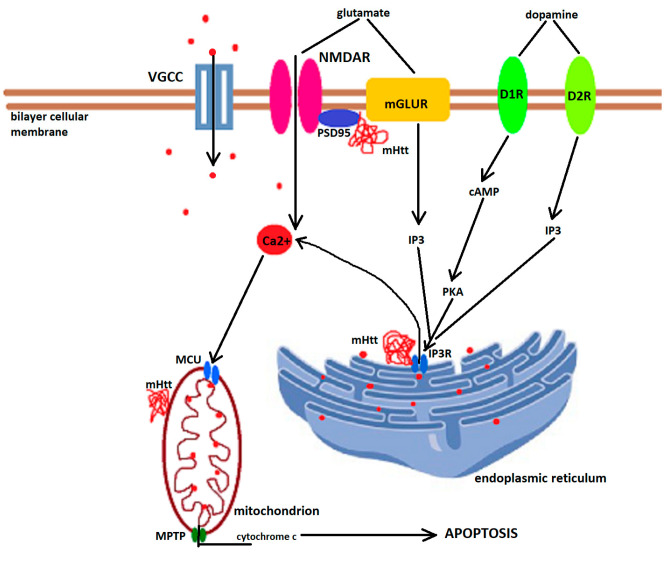
Impaired calcium homeostasis in Huntington’s disease leading to apoptosis. Mutant huntingtin (mHtt) enhances NMDA receptor (NMDAR) function, possibly through decreased interaction with the PDS95 (postsynaptic density 95)/NMDAR complex. Dopamine released by the dopaminergic neurons acts on D1 receptors (D1R), which activate adenyl cyclase, the increased cAMP levels activating phosphokinase A (PKA), which, in turn, stimulates the inositol 1,4,5-phosphate receptors (IP3R), while dopamine acting on D2 receptors directly activate IP3R. IP3R activation leads to release of Ca^2+^ from the endoplasmic reticulum. Excessive cytosolic calcium is taken up by mitochondria through the mitochondrial calcium uniporter (MCU), but will lead to opening of the mitochondrial permeability transition pore (MPTP), especially when mHtt directly associates with mitochondrial membrane proteins. VGCC—L-type voltage-gated calcium channel; red dots—calcium; red whirl—aberrantly folded mutant huntingtin protein.

In addition, mHtt impairs the calcium-buffering capacity of the organelles and leads to increased mitochondrial calcium concentrations, mitochondrial swelling, opening of the MPTP, and release of pro-apoptotic factors, initiating calcium-dependent apoptosis. In buffering intracellular Ca^2+^ concentrations, mitochondria interact closely with the endoplasmic reticulum, which expresses inositol 1,4,5-triphosphate receptors gated by inositol 1,4,5-triphosphate (IP3) and cytosolic calcium. IP3 receptor activity is regulated by many cell surface receptors, including type-5 metabotropic glutamate receptors. The latter modulates IP3 receptor activity through phospholipase C, which hydrolyzes membrane-bound phosphatidylinositol 4,5-biphosphate and releases IP3. By directly binding to the carboxy terminus of the IP3 receptor, mHtt increases IP3 receptor responsiveness, allowing Ca^2+^ release at lower IP3 concentrations [220]. Figure 2 schematically represents the mechanisms of altered cellular calcium homeostasis leading to apoptosis and neurodegeneration.

Notably, striatal MSNs selectively express type-5 metabotropic glutamate receptors. Experiments performed in YAC 128-derived striatal cells showed that IP3 receptor-mediated calcium release from the endoplasmic reticulum elevates the store-operated Ca^2+^ channel (SOC) response [224], leading to dendritic spine loss [220]. Furthermore, by binding to p53 and increasing its transcriptional activity, mHtt upregulates Bcl2-associated X protein (BAX) and p53-upregulated modulator of apoptosis (PUMA), two other pro-apoptotic factors [225]. 

Mutant Htt also significantly impairs mitochondrial morphology and dynamics [226]. Its N-terminus fragment can interact with Mfn2, thereby interfering with the function of Mfn2 in promoting mitochondrial elongation [227], as well as in the interaction of mitochondria with the endoplasmic reticulum in cellular calcium homeostasis [228]. Dysfunctions of Drp1, have also been consistently described in various models of HD, all characterized by fragmentation of mitochondria, which could be corrected by pro-fusion proteins such as Mfn1 and OPA1, or by reducing Drp1 activity and mitochondrial translocation. Translocation of Drp1 to the mitochondria is promoted by calcineurin, which is more active in HD models. Dephosphorylated Drp1 can accumulate and oligomerize on mitochondria, leading to fragmentation of the organelles [229]. The increased activity of calcineurin in HD may be related to decreased expression of endogenous inhibitors [230], increased expression of the phosphatase subunits, or the increase in intracellular calcium concentrations [231], which are in part due to the disturbed function of NMDARs. The disrupted calcineurin activity dephosphorylates and activates the striatal enriched tyrosine phosphatase (STEP), which, in turn, dephosphorylates the GluN2B tyrosine 1472 residue, followed by reduced synaptic NMDAR expression [131]. Research has shown that inhibition of the calcineurin-Drp1 pathway or overexpression of OPA1 can protect against striatal neuronal degeneration. It is still a matter of debate whether the accumulation of mHtt on mitochondria, working as a docking receptor, is sufficient to increase the recruitment of Drp1 on the organelle, or if the interaction of mHtt with Drp1 alters the Drp1 cycling between mitochondria and the cytoplasm. However, by analyzing the mRNA levels of the various genes involved in mitochondrial dynamics, the expression of proteins related to mitochondrial fission, such as Drp1 and Fis1, was found increased, while the expression of fusion proteins (Mfn1, Mfn2, OPA1), was decreased proportionately with the stage of HD [232]. 

Aside from the altered mitochondrial morphology, mitochondrial trafficking is also impaired, along with the trafficking of other vesicles. Both anterograde and retrograde transport are disturbed [233]. The Htt binding protein HAP1 (huntingtin-associated protein 1) interacts with both kinesin and dynein in regulating cargo transport on microtubules. Altered mitochondrial trafficking could be due either to impairment by the protein aggregates or to degradation of Miro as a consequence of organellar dysfunction [234]. Normally, PINK1 and Parkin phosphorylate the GTPase Miro, followed by a detachment of the adaptor protein kinesin from mitochondria and arrest of the organelle. A similar pathway could be triggered in HD. However, the impaired mitochondrial trafficking adds to the disturbed energy metabolism and potentiates neuronal loss in vulnerable brain regions. 

The metabolism of dopamine in the basal ganglia increases oxidative stress. Monoamine oxidases metabolize dopamine into 3,4-dihydroxyphenylacetate (DOPAC) and hydrogen peroxide. DOPAC is further metabolized by catecholamine-O-methyltransferase to homovanillic acid, while hydrogen peroxide reacts with iron to produce hydroxyl radicals [235,236]. In an alternative pathway, dopamine undergoes non-enzymatic auto-oxidation by reacting with molecular oxygen and forms semiubiquinones and superoxide radicals [235]. 

### 5.4. Transcriptional Dysregulation

In situ hybridization studies on postmortem human HD brains showed a decrease in several mRNA species encoding for neurotransmitter receptors and signaling molecules in striatal neurons [135,237]. Subsequently, many studies have shown changes in the levels of mRNA, interactions between mHtt and transcription factors, as well as inhibition of enzymes promoting chromatin remodeling [1]. 

In the regulation of gene expression, the interplay between transcription factors and enzymes in modifying chromatin structure, especially histone acetylation, phosphorylation, methylation, ubiquitination, or sumoylation, is a crucial step. Histone acetyltransferases (HATs) increase gene transcription by adding acetyl groups and opening chromatin architecture, while histone deacetyl transferases (HDACs) remove acetyl groups and repress genes through chromatin condensation [1]. Mutant Htt can interact with several transcription factors and coactivator factors, such as the cAMP response element-binding protein (CREB), CREB binding protein (CBP), the tumor suppressor p53, the transcription elongation regulator 1 (Tcerg1/CA150), or the TATA-binding protein (TBP), as well as other factors [237]. Another mechanism leading to altered transcription is the loss of normal interaction with wild-type huntingtin, as happens with the RE1-silencing transcription factor/neuron-restrictive silencer factor (REST/NRSF) that is physiologically retained by wild-type Htt in the cytoplasm [238], and which, in case of loss of a single allele, is translocated to the nucleus altering the expression of neuronal genes [239]. 

#### 5.4.1. CREB and CBP

CREB (cAMP response element-binding protein) is a member of the leucine zipper family of transcription factors that is phosphorylated at serine 133 and recruits transcriptional co-activators CBP (CREB binding protein) and p300 to activate transcription. In HD, mHtt inhibits the phosphorylation of CREB and the acetyltransferase activity of CBP, which is depleted from its normal nuclear location and sequestrated in polyQ aggregates. In addition, expression of transducers of regulated CREB activity (TORCs) was also found decreased in a HD cellular model, in the striatum of a transgenic mice model, and in postmortem HD striatal tissue [179]. The impaired CREB signaling may lead to impaired BDNF production [240]. 

#### 5.4.2. Peroxisome Proliferator-Activated Receptor Gamma Coactivator-1α

Peroxisome proliferator-activated receptor gamma coactivator-1α (PGC-1α) is a transcriptional coactivator implicated in the regulation of many metabolic processes, including mitochondrial biogenesis. Reduced full-length PGC-1α expression has been repeatedly found in postmortem brain samples from HD patients as well as in transgenic mouse models [241], while levels of the N-truncated splice variant of PGC1α (NT-PGC1α) were found to be upregulated in the human HD brain, and striatal cells of mouse HD models [242]. Mutant Htt represses CRE-mediated transcription of PGC-1α by interfering with the CREB/TAF4 transcriptional pathway in striatal neurons, and experimental restoring of the PGC-1α levels improves neurodegeneration and mHtt aggregation [243]. 

#### 5.4.3. Specificity Protein 1

Specificity protein 1 (Sp1) contains three zinc-finger motifs and a C-terminal domain that synergistically interacts with other transcription factors to control gene expression [244]. Although the role of Sp1 in HD pathogenesis is still controversial, mHtt was shown to inhibit Sp1-dependent transcription in postmortem brain tissues of patients with HD even in the presymptomatic stage [244], while overexpression of Sp1 promotes the expression of wild-type Htt and diminishes the cellular toxic effects of aggregated mHtt [237]. 

#### 5.4.4. Nuclear Factor κ Light-Chain-Enhancer of Activated B Cells

Nuclear factor κ light-chain-enhancer of activated B cells (NF-κB) is a family of DNA-binding proteins acting as transcription factors promoting immune and inflammatory responses, comprising five members: p65/RelA, RelB, p50/NF-κB1, p52/NF-κB2, and c-Rel. RelA is downregulated by mHtt in PC6.3 neuronal cells [245] and activated in astrocytes [246].

#### 5.4.5. Repressor Element 1-Silencing Transcription Factor (REST)

Repressor element 1(RE1)-silencing transcription factor (REST) binds to a DNA element, RE-1/neuron restrictive silencer factor (NRSF), and activates its silencing activity. In the presence of wild-type huntingtin, REST is in the cytoplasm, but mHtt promotes its translocation to the nucleus, where it represses the transcription of target genes [247], including the BDNF gene [248]. As such, molecules able to affect REST/NRSF nuclear translocation, DNA binding, or the formation of the REST/NRSF transcriptional complex, and may serve as potential drug targets in HD.

#### 5.4.6. Other Transcription Factors

Mutant Htt interacts with tumor suppressor p53 leading to phosphorylation at Serine 15 and 46, leading to the expression of apoptotic target genes. The levels of p53 as well as the levels of target genes were found upregulated in striatal and cortical neurons of HD patients [249]. 

Forkhead Box Protein 1 (Foxp1) is highly expressed in the striatum and was found downregulated in HD models and in the caudate nucleus from patients. Although studies performed suggest the possibility of a direct interaction of Foxp1 with mHtt in neurons [250], the role of Foxp1 in glial cells is less clearly defined. 

Heat shock factor 1 (HSF1) is a leucine zipper transcription factor acting as a key regulator of heat shock proteins (HSPs). In the presence of mHtt, the transcription of more than one-third of target genes of HSF1 is impaired [251], significantly interfering with proteostasis. 

#### 5.4.7. Dysregulation of MicroRNAs

Many of the aforementioned gene expression changes are associated with dysregulation of upstream regulators, such as microRNAs (miRNAs), which are approximately 22 nucleotide long molecules belonging to the class of small non-coding RNAs (sncRNAs) [125], which promote degradation or translation repression of target messenger RNAs (mRNAs). Other classes of sncRNAs, such as piwi-interacting RNAs, fragments of small nuclear RNAs, and small nucleolar RNAs can have miRNA-like silencing activity in the regulation of gene expression [252]. Research has shown that in HD, 19 genes had upregulated miRNAs, and 72 genes involved in cellular metabolic processes, neurogenesis, development of the nervous system, transcriptional regulation, or regulation of apoptosis had downregulated miRNAs, among which were HSP40, HSP60, HSP70, or genes encoding for proteins required for ubiquitination, cellular signaling, or epigenetic modifications [252,253]. 

### 5.5. Loss of BDNF Synthesis and Impaired BDNF Transport

BDNF is produced in cortical neurons and transported to medium-sized spiny striatal neurons along the cortico-striatal tract [254], providing strong protection against neuronal death along with glial cell line-derived neurotrophic factor (GDNF) and other neurotrophins such as nerve growth factor, neurotrophin-3 (NT-3), or NT-4/5 [122]. Normally, striatal neurons express very low amounts of BDNF mRNA [254], most of the trophic factors need to be transported from distant sites via axonal transport. Wild-type Htt inhibits the silencing activity of NRSF by sequestering its transcription factor in the cytoplasm, thereby promoting the transcription of the BDNF gene. In contrast, in the presence of mHtt, REST/NRSF accumulates in the nucleus, impairing BDNF gene transcription [255]. Additionally, mHtt interferes with the function of CREB, Sp1, and other transcription factors, and interacts with the glutamine-rich activation domain and the acetyltransferase domain of CBP, causing a more compact structure of chromatin and further impairing BDNF gene transcription [1]. Indeed, low BDNF expression has been reported in the frontal cortex, striatum, substantia nigra, hippocampus, and cerebellum of HD patients and low levels of BDNF protein have been found in the cortex, striatum, and hippocampus of mouse HD models [256]. In addition, phosphodiesterases, enzymes that hydrolyze cyclic nucleotides, are highly expressed in the striatum, and regulate signal transduction via the cAMP/phosphokinase A pathway, leading to inhibition of dopamine signal in the D2 expressing neurons and stimulation of dopaminergic D1 neurons [257]. By regulating protein kinase A and ERK, phosphodiesterases also modulate the transcription and translation of many proteins, including CREB. This makes phosphodiesterase inhibition a potential therapeutic approach in HD [258,259].

Aside from controlling its production, huntingtin also regulates BDNF transport, along with mitochondrial and vesicular transport. Wild-type Htt interacts with HAP1 and recruits glyceraldehyde-3-phosphate dehydrogenase (GAPDH) to provide the necessary energy for transport by producing ATP [260]. The Htt–HAP1 complex binds to kinesin-1 and vesicles, as well as to dynein, serving as an adaptor protein for anterograde transport, or to the dynactin subunit p150glued, mediating retrograde axonal transport [93,261]. The phosphorylation status of Htt at serine 421 by Akt serves as a molecular switch; phosphorylation enhances anterograde transport and release of the vesicular content at axonal terminals [98]. Another huntingtin-interacting protein, HIP1, is pivotal for the assembly and function of the cytoskeletal structures [262]. In HD, GADPH is sequestered into mHtt aggregates and HAP1 binds more tightly to mHtt, thereby reducing the interaction of HAP1/p150 glued with microtubules, likely accounting for the decreased transport of BDNF [2], while HIP1 is more weakly interacting with mHtt, which impair its function [262]. Tubulin acetylation is reduced in HD, leading to reduced binding of motor proteins to microtubules [263], while HAP1 and dynamin are sequestrated into mHtt aggregates [264]. In addition, mHtt activates axonal c-Jun amino-terminal kinase3 via stress-signaling kinase, resulting in kinesin-1 phosphorylation at Serine176 and detachment of kinesin-1 and cargo from microtubules [260]. 

**Figure 3:**
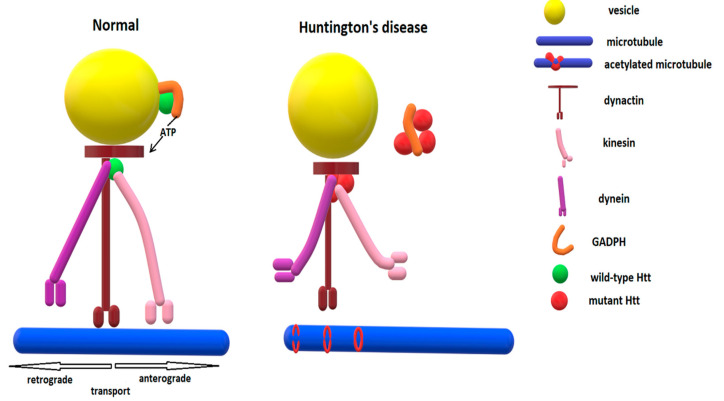
The involvement of Htt in normal vesicular transport and role of mHtt in impaired vesicular transport in HD. Under normal conditions, HTT interacts with the motor proteins dynactin, dynein, and kinesin and recruits GADPH to supply the necessary ATP. In HD, mHtt aggregates sequester GAPDH and motor proteins, and phosphorylates kinesin. Furthermore, microtubules are acetylated by mHtt, which hinders binding of kinesin-1 to the microtubules.

The density of the BDNF receptors, tyrosine receptor kinase B (TrkBs), may also be altered due to transcriptional and trafficking impairments, with low amounts of TrkBs being found in the caudate and cortex of HD brains in post-mortem studies [265]. The retrograde transport of endosomes containing activated TrkBs is reduced in the presence of mHtt, impairing neurotrophin signaling and the Ras/MAPK/ERK1/2 pathway in the striatal dendrites [266]. Defects of Rab11recycling endosomes (Rab11 being a small GTPase important for TrkB distribution in dendrites) may also contribute to impairment of both ligand and receptor dysfunctions of the BDNF/TrkB pathway in HD [267]. Taking into account that BDNF is crucial for the differentiation of striatal neurons and for increasing the length of dendrites and the number of branching points on neurites, cortical BDNF depletion and impaired cortico-striatal transport are key factors in HD pathogenesis. Figure 3 shows schematically the vesicle transport system along microtubules in normal conditions and in Huntington’s disease.

Neurotrophin-3 is another trophic factor widely expressed in the nervous system, which acts by activation of the p75 receptor and of Trk C, B, and A receptors [268], and whose levels are reduced in HD [269]. It has been shown to reduce cellular damage, regulate neuronal plasticity and synapse formation, and promote neuronal regeneration [270], effects which all make neurotrophin-3 an appealing therapeutic target.

### 5.6. Other Disturbances in Signaling

HD progression is accompanied by alterations in neurotransmitter levels and their receptor density in the basal ganglia and midbrain [271], as well as by dysfunction of the neuronal circuit connectivity [121].

Most of the striatal neurons (90–95%) are GABAergic medium spiny neurons (MSNs) receiving mainly glutamatergic cortical and thalamic projections, and projecting to the globus pallidus (GP) pars interna, pars externa, substantia nigra, and thalamus. The remaining 5–10% of neurons are GABAergic and cholinergic interneurons [272]. The MSNs are of two main subtypes: MSNs rich in substance P and dynorphin, enriched in dopaminergic D1 receptors, which project to the GP pars interna and the thalamus, and which are considered to constitute the direct pathway, and MSNs rich in enkephalin, who also express dopaminergic D2 receptors and adenosine A2A receptors, which project to the GP pars externa and belong to the striatal indirect pathway [121]. Any imbalance in the activity of these two pathways results in alterations of movements, as are seen in HD or Parkinson’s disease. However, there are other differences between MSNs in the direct and indirect pathways as well, aside from the dopaminergic receptors they express. In vitro studies have shown differences in resting membrane potential, excitability, threshold for action potential generation [273], and morphologic differences relating to the dendritic surface area [274]. 

A differential pattern of MSN loss has been described. Early in HD, increased glutamate input appears onto MSNs in the direct pathway, likely occurring presynaptically [23] and due to the blocking of endocannabinoid release by MSNs, which eliminates the negative control normally exerted by endocannabinoids on neighboring terminals [275]. Immunohistochemically, enkephalinergic fibers are reduced to about 35% of controls already in stage 1 HD [121]. This correlates clinically with increased stereotypes and chorea. Since GABA normally inhibits dopamine release from nigrostriatal neurons by acting on GABA_B_ receptors [276], loss of MSNs in the direct pathway will lead to an increase in dopamine levels. Loss of GABAergic MSNs also results in a decrease in striatal GABA and a compensatory increase in GABA receptor density in the pars externa of the globus pallidus [277]. The indirect pathway is relatively spared from neurotransmitter imbalance in the early stage of HD [23]. The reason for this differential MSN loss is unclear, but it may be that free or aggregated mHtt might selectively reduce transcription of the enkephalinergic precursor proenkephalin [121]. 

In late HD, the direct pathway no longer shows increased GABA and glutamate synaptic activity [23] due to decreased number of NMDARs and AMPARs, as well as loss of GABAergic MSNs [278], which leads clinically to behavioral hypoactivity. The concomitant alterations in synaptic markers, such as synaptophysin and PSD95, with which Htt colocalizes in the cell, together with the loss of dendritic spines, suggest that MSNs become disconnected [279]. This gradual reduction in glutamatergic synaptic activity also leads to the reduced release of BDNF [248]. The reduced availability of dopamine or D2 receptor stimulation increases GABA release in the indirect pathway, while in the direct pathway, where MSNs do not express D2 receptors, this effect is lacking [23]. 

Purinergic signaling is also disturbed in HD [280]. Adenosine triphosphate and adenosine are the main signaling messengers, which act on P1 and P2 receptors [281]. P1 receptors can be further subdivided into A1, A_2A_, A_2B_, and A3 receptors. A1 receptors interact with D1 receptors, forming heterodimers, whose activation decreases excitatory transmission through neuronal hyperpolarization and calcium channel inhibition, leading to a diminished release of glutamate, dopamine, serotonin, and noradrenaline [282]. A_2A_ receptors form heterodimers with D2 receptors and facilitate the release of glutamate, GABA, acetylcholine, serotonin, and noradrenaline [283]. The number of A1 receptors decreases in the caudate and amygdala of patients with HD, paralleling the severity of the disease, while the loss of A_2A_ receptors is even more dramatic in the caudate, putamen, lateral globus pallidus, olfactory tubercle, and nucleus accumbens in HD, progressing to total loss of A_2A_ receptor binding in late-stage HD [277,284]. P2 purinergic receptors have been less consistently studied in HD, but it appears that cortical and striatal neurons show an increase in P2 receptor protein mRNA levels and the expressed receptors increase their sensitivity to agonists, a situation that favors neuronal apoptosis [285]. 

These complex impairments in neuromediator, co-neuromediator, and neuropeptide balance lead to significant disturbances in brain network connectivity, affecting sensorimotor, executive, attentional, and frontoparietal networks, which translate into motor and non-motor symptoms of HD [286]. 

### 5.7. Astrocytes and Oligodendrocytes in HD

Astrocytes support neural functions by maintaining local environment homeostasis, and envelop neuronal synapses to clear neurotransmitters and ions after synaptic transmission [287]. Failure to clear neuromediators, such as glutamate, can lead to excitotoxicity, while high extracellular potassium levels depolarize neuronal membranes and cause neuronal hyperexcitability [259]. 

Intracellular mHtt aggregates are present in astrocytes as well, although are usually smaller than intraneuronal aggregates [54], suggesting that astrocytes have distinct cleavage mechanisms compared to neurons. A reduction in mHtt levels is able to slow disease progression [288].

K^+^ homeostasis is maintained through mechanisms involving K^+^ channels, gap junctions, and the activity of the Na^+^/K^+^ ATPase [289,290]. Potassium channels can be categorized into four classes: voltage-gated K^+^ channels, two-pore K^+^ channels, Ca^2+^-activated K^+^ channels, and inwardly rectifying K^+^ (Kir) channels [291]. While in neurons, these channels are involved mainly in setting the membrane resting potential; in glial cells, they also play a crucial role in buffering of extracellular K^+^ and interfere with neurotransmitter release, cell proliferation, and apoptosis. While striatal MSNs exhibit a significant reduction in Kir2.1, Kir2.3, and voltage-gated potassium channels leading to increased excitability and altered firing patterns, as well as enhanced NMDAR subunit NR1 expression [292], in glial cells Kir4.1 channel currents were significantly decreased [289]. As such, membrane potential and conductance were found decreased in astrocytes expressing mHtt in mouse models of HD [293]. In fact, the inward flow of K^+^ through Kir channels regulates glial uptake of K^+^ released during neuronal activity, membrane conductance, and sensitivity of glial cells to pH, ATP, or neurotransmitters [294]. 

An important function of astrocytes is to clear excess glutamate via glutamate transporters such as EAAT2 (excitatory amino acid transporter-2) or GLT1 (glutamate transporter-1) [295]. Several studies reported a loss of GLT1 (in mice) and EAAT2 (in humans) both at the mRNA and protein level [296,297], increasing with the severity of HD, which opens the possibility of extrasynaptic NMDAR stimulation through spillover of glutamate to perisynaptic sites [298]. In vitro, co-culturing striatal neurons with mHtt-expressing astrocytes resulted in neuronal death, while in vivo, expression of an N-terminal fragment of mHtt by astrocytes led to reduced GLT1 expression and HD pathology, including astrogliosis [289]. Impairments in astrocyte clearance of excess glutamate could be rescued by restoring Kir4.1 channels or adenoviral-mediated delivery of Kir4.1 into astrocytes [293].

The progressive loss of GLT1 transporters and reduced glutamate clearance also impairs calcium signaling within astrocytes, with reduced spontaneous calcium signals and prolonged action potential-dependent Ca^2+^ signals mediated by metabotropic GluR2/3 receptors [299], which, once stimulated, lead to Ca^2+^ release from intracellular stores. Restoration of Kir4.1 rescued K^+^ and glutamate signaling in astrocytes [293]. An increase in evoked Ca^2+^ level in astrocytes increases sodium pump activity, which further increases extracellular K^+^ concentration [300].

In addition, astrocytes play a key role in cholesterol synthesis in the adult brain [301]. HD associates dysregulated cholesterol metabolism, with lower 24-hydroxy-cholesterol plasmatic levels reported both in HD patients as well as in rodent models [302], both through altered gene expression and at the transcriptional level.

Oligodendrocytes were less extensively studied, but researchers were able to identify myelin damage and breakdown in pre-symptomatic HD patients [303]. The full-length myelin regulatory factor (fMYRF) is self-cleaved to the N-terminal myelin regulatory factor (nMYRF), which is transferred from the ER to the nucleus to promote gene expression. The binding of mHtt to the nMYRF transcription factor in the nucleus impairs these transcriptions, thereby leading to oligodendrocyte dysfunction [304]. In addition, altered PGC-1α expression (through mHtt inhibiting co-binding of CREB and TAF4) reduces the expression of myelin basic protein and causes myelination deficits [305].

### 5.8. Microglial Activation and Neuroinflammation in HD

Neuroinflammation has been increasingly shown to contribute significantly to the pathogenesis of various neurodegenerative processes [306].

Microglia are the resident immune cells of the CNS and constantly monitor the microenvironment of the neurons and glial cells, being able to switch to different activation states through various signaling pathways [307]. Classically, two microglial phenotypes are recognized: the M1 phenotype, which initiates and augments the immune function in the CNS, and the anti-inflammatory M2 phenotype, which contributes to phagocytosis of tissue debris and initiates tissue repair and neural regeneration [307].

Positron emission tomography studies have found significant increases in microglial activation in the striatum of HD patients [308] and in pre-manifest carriers of the HD gene [309], while inflammatory mediators were increased in the striatum, as well as in the cortex and even in the cerebellum of HD patients [310]. Whether this inflammatory response is elicited by degenerating neurons [311] or as a result of mHtt expressed in microglial cells and leading to triggering inflammation through various pathways is still a matter of debate; although, the finding of microglial activation in the cortex and even in the cerebellum, a region generally spared by HD pathology, favors the latter hypothesis.

Mutant Htt promotes the expression of genes encoding for pro-inflammatory cytokines, exaggerating the response of microglia to activating stimuli, which are recognized via nucleotide-binding and oligomerization domain NOD-like receptors (NLRs) and toll-like receptors (TLRs) [312]. In addition, TLR signaling triggers the nuclear factor-κB (NF-κB) pathway and regulates downstream pro-inflammatory cytokine expression, such as interleukin (IL)-1β. IL-6, IL-8, or tumor necrosis factor-α (TNF-α) [313]. An alternative pathway is the kynurenine pathway, the main pathway for nicotinamide adenine dinucleotide formation, in which 3-hydroxyanthranilate 3,4-dioxygenase, strongly expressed in microglia, metabolizes tryptophan to quinolinic acid and 3-hydroxykynurenine. Quinolinic acid acts as an NMDAR agonist and promotes the generation of free radicals [314], contributing to excitotoxic neuronal apoptosis. Cannabinoid receptors (CBRs), a group of G-protein coupled receptors, have also important contributions. CB1 receptors were found abundantly expressed in striatal MSNs and exert neuroprotective effects via inducing expression of BDNF [315], while CB2 receptors, which regulate cell differentiation and survival as well as microglial polarization toward an M2 phenotype through the cyclic AMP protein kinase (PKA), extracellular signal-regulated kinase 1 (ERK1), JUN N-terminal kinases (JNK), and p38 mitogen-activated protein kinase (MAPK) pathways, are expressed mainly in peripheral immune cells but seem a promising therapeutic target, because genetic ablations of these receptors in animal models exacerbated the motor and behavioral impairments [316,317], while administration of phytocannabinoids improved the motor impairments and attenuated neuronal loss in the striatum [318]. In addition, by interacting with the inositol-3-phosphate receptor 1 (IP3R1), by binding to the OMM, or by interacting with voltage-gated Ca^2+^ channels and NMDARs, mHtt impairs the ability of mitochondria to handle increased Ca^2+^ concentrations in microglia as well [319]. The small, prolonged increases in basal intracellular calcium concentrations can trigger the activation of transcriptional programs and change the cellular phenotype. Through the production of nitric oxide, ROS, and cytokines, activated microglia exert effects on macroglia (astrocytes and oligodendrocytes), which, in turn, modulate the activity of microglia via cytokines and neurotransmitters, leading to a complex interaction known as “neuroinflammation” [314]. Molecules released by degenerating neurons, microglia, or astrocytes, generically known as danger-associated molecular patterns (DAMPs), activate astrocytic intracellular signaling pathways such as the Janus kinase/signal transducer and activator of transcription (JAK/STAT) or the NF-κB mitogen-activated protein kinase (MAPK), leading to reactive astrocytes, which increase the expression of cytoskeletal proteins, such as GFAP and vimentin, or other genes involved in the release of cytokines and chemokines [320]. The role of these reactive astrocytes in the progression of HD is still being explored, but it is likely that these cells lose their neuroprotective role as well as their ability to provide support for neurons [321].

## 6. Conclusions

Since the discovery of the mutation of the Htt gene at exon 1 leading to polyglutamine repeats in mHtt as the cause of HD in 1993, the scientific world has struggled to identify the mechanisms through which the mutated protein leads to HD pathology and the clinical manifestations, and find efficient ways to counteract them in order to treat HD. Unfortunately, almost 30 years after that landmark discovery, currently used drugs do not address disease progression and have only limited benefits in controlling motor and behavioral abnormalities.

The brain is a multicellular organism. As such, mHtt-induced cellular dysfunctions are differentially modulated in specific brain regions and cell types. Studies have revealed various spatiotemporal and cell-type-specific pathophysiologic mechanisms in HD. The challenges in HD research are related to the complexity of the pathology from the biochemical level to the system level. Therefore, it is necessary to define how mHtt affects the fate of neurons and glia, and whether therapeutic strategies can selectively modulate the function of the striatal neurons while preventing adverse behavior of glial cells. The identification of blood cell-derived markers for HD that can mimic the brain molecular pathology could facilitate early diagnosis.

The encouraging results obtained in animal models with drugs targeting the various pathogenic molecular mechanisms highlighted above open the possibility of novel therapeutic approaches. Furthermore, gene therapy is a rapidly expanding area. Nonetheless, the exact timing when these novel therapeutic approaches are most efficient requires further research.

## Data Availability

Data sharing not applicable.
